# Post-neoadjuvant cellular dissociation grading based on tumour budding and cell nest size is associated with therapy response and survival in oesophageal squamous cell carcinoma

**DOI:** 10.1038/s41416-019-0623-2

**Published:** 2019-11-06

**Authors:** Moritz Jesinghaus, Melanie Boxberg, Dirk Wilhelm, Stefan Münch, Hendrik Dapper, Michael Quante, Christoph Schlag, Sebastian Lange, Jan Budczies, Björn Konukiewitz, Martin Mollenhauer, Anna Melissa Schlitter, Karl Friedrich Becker, Marcus Feith, Helmut Friess, Katja Steiger, Stephanie E. Combs, Wilko Weichert

**Affiliations:** 1 Institute of Pathology, Technical University Munich, Munich, Germany; 2 German Cancer Consortium (DKTK), Partner Site Munich, Munich, Germany; 3 German Cancer Consortium (DKTK), Partner Site Munich, Institute for Translational Cancer Research, Munich, Germany; 4 Department of Surgery, Klinikum rechts der Isar, Technical University Munich, Munich, Germany; 5 Department of Radiation Therapy, Klinikum rechts der Isar, Technical University Munich, Munich, Germany; 6 II Medizinische Klinik, Klinikum rechts der Isar, Technical University Munich, Munich, Germany; 7 Institute of Pathology, University Hospital Heidelberg, Heidelberg, Germany; 8 Institute of Innovative Radiotherapy (iRT), Helmholtz Center Munich, Munich, Germany

**Keywords:** Prognostic markers, Oesophageal cancer

## Abstract

**Background:**

Cellular Dissociation Grade (CDG) composed of tumour budding and cell nest size has been shown to independently predict prognosis in pre-therapeutic biopsies and primary resections of oesophageal squamous cell carcinoma (ESCC). Here, we aimed to evaluate the prognostic impact of CDG in ESCC after neoadjuvant therapy.

**Methods:**

We evaluated cell nest size and tumour budding activity in 122 post-neoadjuvant ESCC resections, correlated the results with tumour regression groups and patient survival and compared the results with data from primary resected cases as well as pre-therapeutic biopsies.

**Results:**

CDG remained stable when results from pre-therapeutic biopsies and post-therapeutic resections from the same patient were compared. CDG was associated with therapy response and a strong predictor of overall, disease-specific (DSS) and disease-free (DFS) survival in univariate analysis and—besides metastasis—remained the only significant survival predictor for DSS and DFS in multivariate analysis. Multivariate DFS hazard ratios reached 3.3 for CDG-G2 and 4.9 for CDG-G3 neoplasms compared with CDG-G1 carcinomas (*p* = 0.016).

**Conclusions:**

CDG is the only morphology-based grading algorithm published to date, which in concert with regression grading, is able to contribute relevant prognostic information in the post-neoadjuvant setting of ESCC.

## Background

Oesophageal squamous cell carcinoma (ESCC) represents the most common subtype of oesophageal cancer worldwide. ESCC is often associated with an aggressive disease course, and a substantial percentage of patients are in locally advanced clinical stages at the time of initial diagnosis.^1–4^ In the absence of distant metastases, neoadjuvant chemoradiation with consecutive tumour resection is generally the treatment of choice for patients with operable tumours and a locally advanced clinical tumour stage (cT3/4) or clinical suspicion of nodal metastases (cN +).^5–9^

In the neoadjuvant therapy setting, patient prognosis and consequently the selection of postoperative treatment modalities are mainly determined by postoperative histopathologic staging and (to a lesser extent) histopathologic tumour regression grading.^10–14^ Classical WHO-based histopathologic grading of ESCC, which is based on the morphological factors keratinization, nuclear pleomorphism and mitotic activity,^15–19^ is not applied after neoadjuvant therapy, not only because chemoradiation is known to profoundly influence critical factors of this grading system such as nuclear size^20,21^ but also because its prognostic significance is highly controversial even for primary resected ESCC and de facto non-existent after neoadjuvant therapy.^16–18^

Recently, a novel grading approach termed Cellular Dissociation Grade (CDG) based on the evaluation of tumour budding and cell nest size, which are histologic factors that measure the extent of cellular dissociation either from a quantitative (tumour budding) or a qualitative (cell nest size) angle, was proposed for squamous cell carcinomas from various anatomic sites.^22–24^ In ESCC, Cellular Dissociation Grading has been shown to be a stage-independent predictor of the disease course when measured in pre-therapeutic biopsies^25^ as well as in primary resection specimens.^26^ However, the feasibility and the prognostic significance of the application of the Cellular Dissociation Grade in ESCC after neoadjuvant treatment has not been investigated so far.

In order to probe for the transferability of the Cellular Dissociation Grade to the post-neoadjuvant treatment setting in ESCC, we investigated tumour budding and cell nest size in a cohort of 122 ESCC resection specimens after neoadjuvant chemoradiation and correlated the results with postoperative pathological staging (ypT, ypN and ypM), histopathologic tumour regression scoring and overall-, disease-free- as well as disease-specific survival. Furthermore, we compared the Cellular Dissociation Grade derived from resection specimen after neoadjuvant treatment with data from pre-therapeutic biopsies of the same patients^25^ and with data from a cohort of primary resected ESCC.^26^

## Materials and methods

### Cohort

Our cohort comprised resection specimens from 122 patients suffering from ESCC, who underwent resection after neoadjuvant chemoradiation. Patients were surgically treated between 1991 and 2016 at the University Hospital Rechts der Isar of the Technical University Munich. Mean patient age was 57 years (range: 30–80); male patients were more common than female ones (95/122; 77.9%). Neoadjuvant chemotherapy combined with radiation therapy was administered in a synchronous fashion according to the guidelines in place during the time of treatment. The exact chemoradiation scheme was available for 66 of the 122 patients (54%). Radiation dose ranged from 28.8 to 60 Grey (Gy; mean: 43.3 Gy; median: 45 Gy). Sixty-three of the 66 patients received a dose of 40 Gy or more. The chemotherapy scheme was Platin-based in 80% of these patients (*n* = 53), the remaining patients (*n* = 13, 20%) received 5-fluorouracil (5-FU) monotherapy. Of the patients with Platin-based chemotherapy, Cisplatin monotherapy (*n* = 25, 47%) was the most common therapy approach followed by Oxaliplatin combined with 5-FU (*n* = 11, 21%) and Carboplatin combined with Paclitaxel (*n* = 7, 13%). The time span from the date of the initial preoperative diagnosis (as a surrogate time point for the date of initiation of chemoradiation) to surgical resection was available for 113 of the 122 patients (93%) and ranged from 51 to 168 days (mean: 95.7 days). The general surgical procedure applied for all patients with ESCC was abdomino-thoracal resection after Ivor-Lewis,^27^ which is the standard surgical procedure for oesophageal cancer at our hospital.

For 42 (34.4%) patients, additional data on the Cellular Dissociation Grade from pre-therapeutic biopsy specimen of the same case were available.^25^ A cohort of 135 primary resected ESCCs for which the prognostic impact of the Cellular Dissociation Grade was analysed in a previous study was selected as a second control group.^26^

Pre-therapeutic staging data were available for 120/122 patients (cT2: *n* = 6, cT3: *n* = 107 and cT4: *n* = 7; cN0: *n* = 23, cN1: *n* = 97). Post-neoadjuvant ypTNM data were available for all patients. Histopathological assessment after chemoradiation of the respective resection specimen resulted in 47 patients with carcinomas in a rather early local stage (ypT1/2; 38.5%; ypT1: *n* = 12, ypT2 *n* = 35) and 75 patients with locally advanced tumours (ypT3/4; 71.5%; ypT3: *n* = 71, ypT4: *n* = 4). Vital lymph node metastases were detected in 47 patients (38.5%; ypN0: *n* = 75, ypN1: *n* = 45 and ypN2: *n* = 2). In total, 95 patients (77.9%) died during follow-up, 86 deaths were tumour specific. A relapse of the disease was noted in 92 (75.4%) patients during follow-up (Table 1). An approval for this study was obtained from the Ethics Committee of the Faculty of Medicine of the Technical University Munich (503/16S).

**Table 1:** Association of clinicopathological factors, cell nest size, tumour budding activity and Cellular Dissociation Grade with survival parameters in univariate survival analysis

	Overall	Events (OS)	Mean overall survival	*p*-value	Events (DSS)	Mean disease-specific survival	*p*-value	Events (DFS)	Mean disease-free survival	*p*-value
*Age*										
Median and below	59	43	55.2	*0.05*	37	61.0	*0.022*	42	52.6	*0.45*
Above median	63	52	48.0		49	52.8		50	45.2	
*Sex*										
Male	95	74	58.8	*0.80*	65	69.0	*0.539*	70	59.9	*0.399*
Female	27	21	41.6		21	41.6		22	32.1	
*ypT*										
1	12	8	63.4	*0.16*	7	69.9	*0.199*	8	59.4	*0.434*
2	35	25	56.2		24	58.7		26	50.3	
3	71	58	37.6		53	40.2		56	35.4	
4	4	4	66.9		2	95.8		2	95.8	
*ypN*										
0	75	55	71.8	*0.09*	48	84.5	*0.065*	51	73.9	*0.041*
1	45	38	33.7		36	36.3		39	*29.9*	
2	2	2	26.2		2	26.2		2	*19.9*	
*ypM*										
0	117	91	57.9	*0.125*	82	66.1	*0.094*	87	57.8	*0.02*
1	5	4	14.8		4	14.8		5	12.4	
*Regression grade* (*Becker et al.*)										
1B	51	34	58.0	*0.026*	29	65.0	*0.013*	32	56.4	*0.012*
2	22	16	47.3		16	47.3		17	*44.1*	
3	49	45	40.5		41	46.2		43	*38.8*	
*Tumour budding (1 HPF)*										
None	15	7	77.9	*0.025*	4	96.1	*0.003*	5	83.9	*0.001*
Low (2–4 buds)	49	42	52.9		37	62.5		49	55.7	
High ( ≥5 buds)	58	46	39.3		45	40.6		58	33.9	
*Cell nest size*										
>15 cells	11	5	77.7	*0.085*	3	95.4	*0.010*	3	95.7	*0.01*
5–15 cells	4	2	74.5		1	95.9		2	67.2	
2–4 cells	19	15	59.6		12	64.7		13	54.3	
Single cells	88	73	50.8		70	53.4		74	45.3	
*Cellular Dissociation Grade*										
CDG-G1 (score 2–3)	15	7	77.9	*0.038*	4	96.1	*0.004*	5	83.9	*0.003*
CDG-G2 (score 4–5)	19	16	57.7		13	62.3		14	52.3	
CDG-G3 (score 6–7)	88	72	51.0		69	53.6		73	45.5	

Italics values indicate statistical significance *p*-values

### Histologic evaluation

Haematoxylin and eosin-stained slides from resection specimens of oesophageal squamous cell carcinoma after chemoradiation were evaluated by an experienced gastrointestinal pathologist (M.J.; observer 1), who was blinded for clinicopathological data and the disease course. Interobserver reproducibility was probed by randomly selecting 45 cases from the cohort, that were evaluated by a second experienced pathologist (M.B., observer 2), fully blinded to the results of the initial evaluation by observer 1.

Furthermore, all carcinomas were divided into keratinizing, non-keratinizing or basaloid neoplasms. Tumour regression was scored in analogy to the regression score proposed by Becker et al.,^13,14^ subclassifying neoadjuvantly treated ESCCs in complete responders (grade 1A; complete regression), subtotal responders (grade 1B; subtotal regression, <10% vital tumour), partial responders (grade 2; marked regression, between 10 and 50% vital tumour) and nonresponders (grade 3; slight/no regression, >50% vital tumour). Tumours showing complete regression could not be included in the cohort for the obvious reason that in these cases no gradeable tumour was present anymore.

**Fig. 1:**
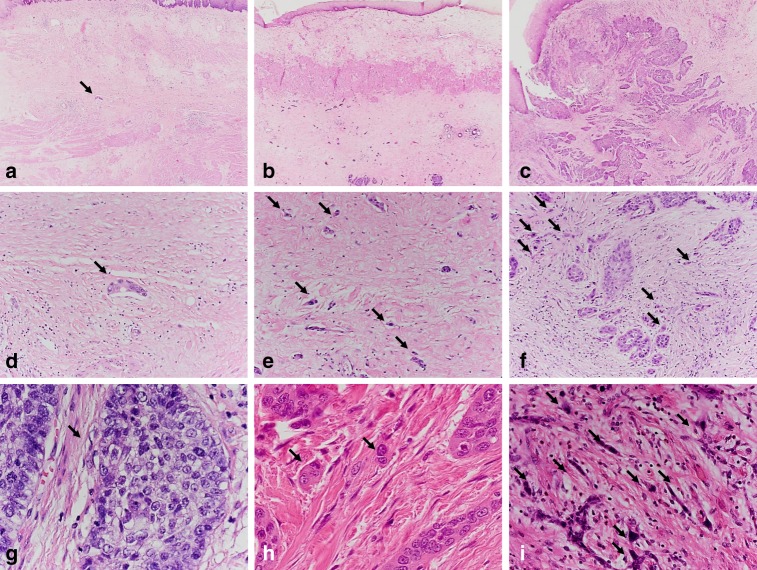
Scanning magnification of an ESCC with a subtotal response (regression grade 1B; <10% vital tumour cells) to neoadjuvant therapy (**a**) showing a good differentiation (CDG-G1) according to the Cellular Dissociation Grade with only a singular cell nest (arrow) within the tumour bed composed of >5 tumour cells (no tumour budding, intermediate cell nest size), which is also shown in higher magnification (**d**; ×20). Scanning magnification of an ESCC with a marked response (regression grade 2; >10%, <50% vital tumour cells) to neoadjuvant therapy (**b**) showing a poor differentiation according to the Cellular Dissociation Grade (CDG-G3), with numerous small tumour cell complexes <5 cells (high tumour budding activity; arrows) within the tumour bed and multiple foci of single-cell invasion (smallest cell nest size), which are shown also in a higher magnification (**e**; ×20). Scanning magnification of an ESCC with a poor response (regression grade 3; >50% vital tumour cells) to neoadjuvant therapy (**c**) showing a poor differentiation according to the Cellular Dissociation Grade (CDG-G3), with numerous small tumour cell complexes <5 cells (high tumour budding activity; arrows) within the tumour bed and multiple foci of single-cell invasion (smallest cell nest size), which are shown also in a higher magnification (**f**). High magnification (×40) of tumour budding and cell nest size in 1 HPF: well-differentiated ESCC (CDG-G1; **g**) without tumour budding and with large cell nest size (>15 tumour cells; arrow). Moderately differentiated ESCC (CDG-G2; **h**) with low tumour budding (2–4 tumour buds per 1 HPF) and with small cell nest size (2–4 tumour cells; arrows) but without single-cell invasion. Poorly differentiated ESCC (CDG-G3; **i**) with high tumour budding (≥5 tumour buds per 1 HPF; arrows) and single-cell invasion (arrows)

The dissociation of small tumour clusters consisting of <5 neoplastic cells into the peritumoural stroma in an invasive fashion was defined as “tumour budding”. Tumour budding activity was investigated throughout the whole slide and scored within the high- power field showing the highest budding activity. The visible area in ×40 magnification was defined as a high-power field (HPF). The field diameter for one HPF (0.55 mm) was determined through division of the field number (22 ×) by the objective magnification (× 40). Low tumour budding activity was stated if 1–4 and high tumour budding activity was stated if ≥5 budding foci were identified (Fig. 1).

As tumour budding activity, cell nest size was evaluated in analogy to the algorithm from previous studies.^23,26,28^ Cell nests were defined as large if the smallest cell nest within a tumour comprised >15 and as intermediate if it consisted of 5–15 tumour cells, respectively. Small cell nest size was scored if the smallest nest consisted of 2–4 tumour cells. Singular, discohesive tumour cells without nested architecture were classified as single-cell invasion. For every ESCC, the smallest identifiable cell nest size within the resection specimen was reported (Fig. 1). For example, in an ESCC after chemoradiation mainly comprising small cell nests with a single focus of single-cell invasion, the respective cell nest size was classified as single-cell invasion.

### Composition of the Cellular Dissociation Grade

We established a grading system that we termed “Cellular Dissociation Grade” (CDG) by using a combined score incorporating both parameters, tumour budding and cell nest size. The modified grade is in analogy to our previous work of primary resected ESCC, that evaluated the extent of both factors in 10 HPFs.^23,26,28^ In order to translate this grading approach to ESCC after neoadjuvant therapy (10 HPFs are not feasible in some tumours with subtotal response), all carcinomas were evaluated for tumour budding within one HPF, reporting the HPF that showed the highest budding activity. In addition, the whole tumour was evaluated for the smallest cell nest size. This approach is in complete analogy to the evaluation algorithm applied in the ESCC biopsy setting.^25^ A score of 1 received tumours without budding activity, tumours with low budding activity (<5 buds per HPF) were assigned a score of 2 and tumours with high budding frequency (≥5 buds per HPF) received a score of 3. Regarding cell nest size, tumours with large cell nests (>15 cells) received 1 point, while tumours with intermediate (5–15 cells) and small (2–4 cells) nests were scored with 2 and 3 points, respectively. ESCCs with single-cell invasion were scored with 4 points. The sum of both variables resulted in a final grading score ranging from 2 to 7. By using this score, we composed a Cellular Dissociation Grade algorithm for ESCC after neoadjuvant treatment, which is generally identical to the grading algorithm proposed for primary resected ESCC and pre-therapeutic biopsy specimens.^25,26^ CDG-G1 (well-differentiated) ESCC had a score ranging from 2 to 3, CDG-G2 (moderately differentiated) neoplasms had a score ranging from 4 to 5 and CDG-G3 (poorly differentiated) carcinomas had a score from 6 to 7. The algorithm for Cellular Dissociation Grading of ESCC after neoadjuvant treatment is summarised in Table 2.

**Table 2:** Algorithm to determine the Cellular Dissociation Grade derived from tumour budding activity (1–3 points) and cell nest size (1–4 points) in ESCC after neoadjuvant treatment

Cellular Dissociation Grade for oesophageal squamous cell carcinoma after neoadjuvant treatment	
*Tumour budding activity/1 HPF*	
No budding	1
<5 budding foci	2
≥5 budding foci	3
*Smallest cell nest size*	
>15 cells	1
5–15 cells	2
2–4 cells	3
Single-cell invasion	4
*Cellular Dissociation Grade*	Total score
Well differentiated (CDG-G1)	2–3
Moderately differentiated (CDG-G2)	4–5
Poorly differentiated (CDG-G3)	6–7

### Statistics

Statistical analysis was performed by using the Statistic Package for Social Sciences 25.0 (SPSS, Chicago, IL, USA). Comparisons of tumour budding, cell nest size and Cellular Dissociation Grade between primary resections, resections after neoadjuvant therapy and pre-therapeutic biopsies were calculated with Mann–Whitney U and Wilcoxon tests. Associations of histopathological characteristics with clinicopathological parameters were calculated with χ^2^ (chi-square) test as well as χ^2^ test for trends. Survival analyses were performed by using the Kaplan–Meier method; a log-rank test was used to investigate the significance of differences in survival probabilities. Multivariate survival analysis was performed by using the Cox proportional hazard model. *P*-values ≤ 0.05 were considered significant. As only planned hypothesis testing was performed, no corrections for multiple testing were necessary in this study.^29^

## Results

### Distribution of histomorphological features

In our neoadjuvantly treated cohort, keratinizing tumours (63/122, 51.6%) were more frequent than non-keratinizing cancers (40/122, 32.8%), 19 ESCCs showed a basaloid morphology (19/122, 15.6%). Regression grades according to Becker^13,14^ were grade 3 (slight/no regression, more than 50% of the tumour still viable) in 49 out of 122 cases (40.2%), grade 2 (marked regression, between 10 and 50% of the tumour viable) in 22/122 cases (18%) and grade 1B (subtotal regression, <10% of viable tumour) in 51/122 cases (41.8%).

Tumour budding activity (per 1 HPF) was distributed as follows: 15 cases (12.3%) had no budding activity, 49 tumours showed low budding (2–4 buds per one HPF) activity (40.2%) and 58 cancers had high budding (5 or more buds per one HPF) activity (47.5%). The analysis of the minimal cell nest size revealed large cell nests (>15 tumour cells) in 11 cases (9%), intermediate nests (5–15 tumour cells) were present in 4 tumours (3.2%) and 19 cases (15.6%) showed small nests (2–4 tumour cells). In the vast majority of cases (88/122, 72.1%), infiltrative clusters of singular tumour cells were observed and these cases were therefore classified as having single-cell invasion (Table 1). Budding activity and cell nest size in neoadjuvantly treated oesophageal carcinoma was correlated (*r* = 0.717, *p* < 0.001), as seen previously in a primary resected^26^ and a biopsy cohort.^25^

The calculation of the Cellular Dissociation Grade on the basis of the sum of the scoring points attributed for tumour budding (1–3 points) and cell nest size (1–4 points) for each specimen resulted in 15 (12.3%) CDG-G1 (2–3 points) neoplasms, 19 (15.6%) CDG-G2 (4–5 points) tumours and 88 (72.1%) CDG-G3 (6–7 points) carcinomas. Figure 1 illustrates different regression grades, tumour budding and cell nest size as well as the differentiation grades according to the Cellular Dissociation Grade.

### Tumour budding, cell nest size and cellular dissociation grade in dependence of neoadjuvant therapy

We compared the frequency of tumour budding, cell nest size and Cellular Dissociation Grade in our neoadjuvantly treated cohort with our own previously published distribution data in a primary resected cohort (*n* = 135) of almost equal size.^26^ Tumour budding was significantly higher in the neoadjuvantly treated cohort than in the primary resected population (*p* = 0.004). The same was true for cell nest size (*p* < 0.001); specifically the number of cases that showed single-cell invasion was substantially increased. Consequently, overall the group of neoadjuvantly treated cases showed significantly higher Cellular Dissociation Grades than the group of primary resected cancers (*p* < 0.001). This difference was likely due to the fact that full responders (ypT0)—who are not present in our neoadjuvantly treated cohort since no evaluable tumour is left—often fall into the CDG-G1/G2 category.

In the subset of patients where the exact chemoradiation scheme was available, neither the type of chemotherapy nor the radiation dose were statistically associated with the post-neoadjuvant Cellular Dissociation Grades (*p* > 0.2, respectively). The time span between the initial diagnosis and the surgical resection was also not correlated with post-neoadjuvant Cellular Dissociation Grade (*p* > 0.5).

**Fig. 2:**
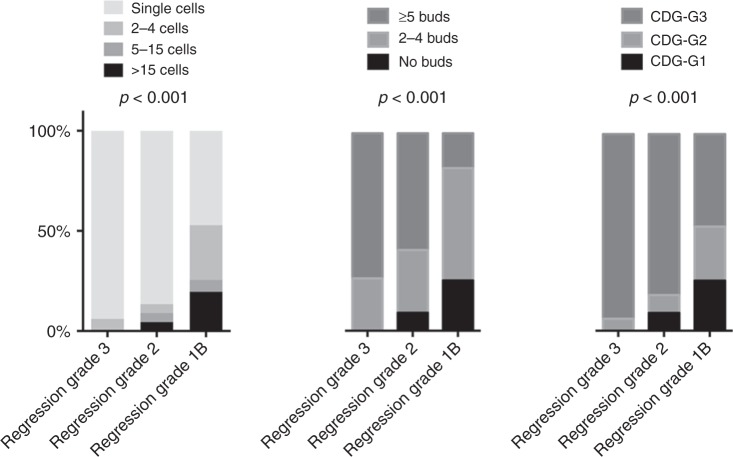
Association of Cellular Dissociation Grade with tumour regression in post-neoadjuvant resection specimens from oesophageal squamous cell carcinoma. Note the higher frequency of poorly differentiated ESCCs (CDG-G3) in the nonresponder subgroup (regression grade 3) compared with tumours with marked- (regression grade 2) or subtotal (regression grade 1B) response

In 42 cases of our cohort, we had tumour budding and cell nest size information, as well as Cellular Dissociation Grades not only from the resection specimens after chemoradiation but also from the respective pre-therapeutic biopsies from the same patients. The data from the biopsies were part of a previous study published by us.^25^ When we compared the pre- and post-treatment distributions of tumour budding, cell nest size and Cellular Dissociation Grade, no significant differences for all three parameters were noted (*p* = 0.707 for tumour budding, *p* = 0.790 for cell nest size and *p* = 0.920 for Cellular Dissociation Grade, Fig. 2).

### Interobserver variance for Cellular Dissociation Grade

Interobserver analysis of 45 randomly selected cases revealed a high reproducibility for the Cellular Dissociation Grade between two independent pathologists (Kappa–Cohens value: 0.89; Supplementary Table 1) fully blinded for the diagnosis of the other pathologist, proving that the generally high reproducibility for the novel grading system previously shown in other clinical settings^30^ also holds true in this type of specimen.

### Correlation of tumour budding, cell nest size and Cellular Dissociation Grade with regression grade

Both tumour budding as well as cell nest size were strongly associated with the extent of tumour regression induced by neoadjuvant therapy. The smallest cell nest sizes and the highest budding activity was observed in those tumours that showed minimal to no regression (more than 50% viable tumour) as response to neoadjuvant treatment. In contrast, those cases with strong regression (<10% viable tumour) usually did not show single-cell invasion and strong budding activity (*p* < 0.001 for both comparisons). As a consequence, composite Cellular Dissociation Grade was also associated with regression, minimally regressive tumours were significantly less differentiated than tumours with strong regression (*p* < 0.001, Fig. 2). When we correlated the Cellular Dissociation Grade from pre-therapeutic biopsies^25^ that was available for 42 patients in our cohort, with the histopathologic response after chemoradiation, it became apparent that also in this setting a lower differentiation according to the Cellular Dissociation Grade was associated with an inferior response to neoadjuvant treatment (*p* = 0.042).

### Correlation of the Cellular Dissociation Grade with clinicopathological parameters including stage

When we correlated cell nest size, tumour budding and Cellular Dissociation Grade with staging data, a higher budding activity, smaller cell nest size and poorer differentiation was significantly associated with higher ypN stage (*p* < 0.05) but not with age, sex, ypT or ypM (Supplementary Table 2). Furthermore, lymphatic vessel invasion (L, *p* = 0.09), but not perineural invasion (Pn) or venous invasion (V) was associated with a poorer differentiation according to CDG.

### Correlation of tumour budding, cell nest size and Cellular Dissociation Grade with survival

**Fig. 3:**
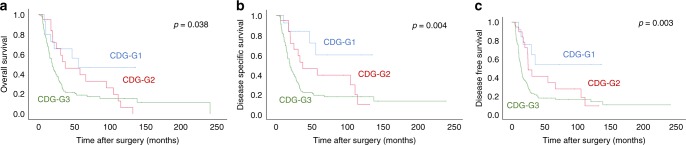
Association of the Cellular Dissociation Grade with overall (**a**), disease-specific (**b**) and disease-free (**c**) survival in resected ESCC after neoadjuvant treatment

High tumour budding activity as well as small cell nest sizes were associated with overall (OS), disease-specific (DSS) and disease-free (DFS) survival (Table 1). The association was stronger for budding activity (*p* = 0.025, 0.003 and 0.001, respectively) than for cell nest size (*p* = 0.085, 0.010 and 0.010, respectively, Supplementary Fig. 1); the raw scores derived from the sums of both factors are shown in Supplementary Fig. 2. Consequently, composite Cellular Dissociation Grade as determined on post-neoadjuvant samples had strong impact on all three survival parameters, as well. Patients with well- differentiated tumours (CDG-G1) had an overall (disease-free) survival of 77.9 (83.9) months, while patients with moderately differentiated tumours (CDG-G2) survived 57.7 (52.2) months and patients with poorly differentiated tumours (CDG-G3) showed survival times of 51.0 (45.5) months, these survival differences were significant (*p* = 0.038 and 0.003, respectively; Fig. 3), making the Cellular Dissociation Grading system the first morphology-based prognostic grading system working on tissue after neoadjuvant therapy in oesophageal squamous cell carcinoma.

When we performed a multivariate analysis under inclusion of ypT, ypN, ypM, regression grade and Cellular Dissociation Grade, the only prognostically relevant factors that emerged were ypM and Cellular Dissociation Grade. Patients whose tumours were poorly (moderately) differentiated had a hazard ratio of 4.99 (3.35) for DFS, when compared with their well-differentiated counterparts (*p* = 0.016, Table 3). For DSS, the same significant associations were noted (*p* = 0.029, Supplementary Table 3), while for OS Cellular Dissociation Grade narrowly failed to reach statistical significance in multivariate analysis (*p* = 0.150, Supplementary Table 4).

**Table 3:** Association of Cellular Dissociation Grade with disease-free survival in multiparametric regression analysis

	HR (DFS)	Lower CI (95%)	Upper CI (95%)	*p*-value
*Age (per year)*				**0.205**
Median and below	1.00			
Above median	1.72	0.991	1.04	
*ypT stage*				**0.698**
1	1.00			
2	1.14	0.49	2.60	
3	1.42	0.58	3.43	
4	0.73	0.14	3.93	
*ypN stage*				**0.655**
0	1.00			
1	1.25	0.78	1.99	
2	1.18	0.27	5.10	
*ypM stage*				**<0.001**
0	1.00			
1	7.40	2.50	21.91	
*Regression grade*				**0.521**
1B	1.00			
2	0.93	0.47	1.83	
3	1.29	0.69	2.40	
*Cellular Dissociation Grade*				**0.016**
CDG-G1	1.00			
CDG-G2	3.35	1.09	10.32	
CDG-G3	4.99	1.65	15.09	

Bold values indicate statistical significance *p*-values

Although there is a certain overlap with nodal positivity, we additionally performed an exploratory multivariate analysis under inclusion of lymphatic vessel invasion (L0/L1), this resulted in an even stronger p-value for CDG for DFS (*p* = 0.006) and DSS (*p* = 0.016).

Interestingly, when we subdivided our cohort according to regression grade and stratified the subgroups for Cellular Dissociation Grade,^13,14^ we found that the prognostic effect of Cellular Dissociation Grade on survival was also visible in the group of tumours with subtotal regression (regression grade 1B, Supplementary Fig. 3). Tumours with a poor differentiation in this subgroup showed a significantly shortened DSS (*p* = 0.034) and DFS (*p* = 0.047) compared with well/moderately differentiated tumours (grouped because of low numbers). A clear impact on OS, however, was not observed. Regression grade 3 tumours were almost exclusively poorly differentiated (46 out of 49 cases, Supplementary Table 2), which renders a statistical analysis impossible. The number of tumours in the regression grade 2 group was too small to detect any meaningful differences (*n* = 22).

## Discussion

In this study, we investigated the transferability of Cellular Dissociation Grading based on tumour budding and cell nest size, which has already been established in primary resection specimens^26^ as well as in pre-therapeutic biopsies of oesophageal squamous cell carcinoma,^25^ to the neoadjuvant setting by evaluating these factors in a large cohort of 122 ESCC specimens resected following neoadjuvant treatment. We were not only able to confirm the prognostic power of the Cellular Dissociation Grade in post-neoadjuvant resection specimens, but were also able to demonstrate that the Cellular Dissociation Grade remained the only prognostic factor for DSS/DFS besides the presence of distant metastases in a multivariate analysis incorporating postoperative pathologic staging (ypT, ypN and ypM), and tumour regression.^13,14^

The proposed grading is reproducible in this setting, which is in line with previous observations not only in biopsy specimens of ESCC but also in other tumour entities with squamous morphology.^22,25^

Combined platinum-based chemotherapy and radiation^9^ with consecutive surgical resection reflects the common therapy approach for locally advanced but still operable ESCC in the absence of distant metastases.^5–8^ In the postoperative setting of neoadjuvantly treated ESCC, histopathologic stage and tumour regression are considered to be among the strongest factors that determine the further course of the disease.^10–14^ Nevertheless, a considerable amount of ESCC patients with a partial or even subtotal response to neoadjuvant treatment suffer from disease relapse and consecutively die of the disease,^12^ underlining the need for additional biomarkers in addition to staging and tumour regression grading that allow for an improved prognostic patient stratification in the postoperative setting.

Classical histopathologic grading of ESCC is currently performed in accordance with the algorithm of the WHO classification of tumours of the digestive system^15^ and relies on traditional histomorphologic parameters such as nuclear pleomorphism, mitotic activity and degree of keratinization. In neoadjuvantly treated ESCC, WHO-based grading is usually omitted, not only because chemoradiation is known to considerably disarray relevant factors to this algorithm such as nuclear size/pleomorphism^20,21^ but also because it is almost irrelevant for clinical decision-making even in primary resected ESCC, due to its at best-limited prognostic significance.^16–19^

Tumour budding, a histologic parameter that measures the quantitative extent of cellular dissociation of a neoplasia, has been identified as a highly prognostic factor in ESCC^31–36^ and other squamous cell carcinoma entities.^37,38^ Our proposed grading algorithm that has been shown to nicely discriminate prognostic subgroups in primary resected ESCCs^26^ as well as in pre-therapeutic ESCC biopsies^25^ and several other squamous cell carcinoma entities,^23,24,39^ pairs up tumour budding with cell nest size, a second parameter measuring cellular dissociation from a more qualitative angle. Therefore, our novel grading approach has been termed “Cellular Dissociation Grade”. While we already demonstrated preoperative Cellular Dissociation Grading performed on biopsies to be a strong pre-treatment predictor for ESCC survival independent from clinical staging in a previous study,^25^ the prognostic implications of Cellular Dissociation Grade in the post-neoadjuvant setting of ESCC have not been investigated until now. In this study of 122 post-neoadjuvant ESCC resection specimens, the Cellular Dissociation Grade retained its prognostic power in all survival comparisons, making the Cellular Dissociation Grade—to our knowledge—the first fully and solely morphology-based prognostic system working on tissue after neoadjuvant therapy in ESCC. The prognostic relevance of the Cellular Dissociation Grade is further underlined by our multivariate analysis incorporating the only currently known prognostic factors in the postoperative setting (ypTNM, tumour regression) of ESCC after chemoradiation, where Cellular Dissociation Grade remained the only multivariate prognosticator for DSS/DFS besides distant metastases. Even in the subset of ESCC with high tumour regression (<10% residual tumour), tumour remnants showing a poor differentiation according to the Cellular Dissociation Grade were predictive of shortened DSS/DFS, which means that the grading system is able to further prognostically stratify patients even when they showed a good initial response to neoadjuvant treatment.

Besides the retained impact of Cellular Dissociation Grade on survival even after neoadjuvant therapy, specifically the association of the grading system with different therapy responder groups is very interesting. When we correlated tumour differentiation according to the Cellular Dissociation Grade with tumour regression grades, almost all of the nonresponders showed a poor differentiation, while well-differentiated and moderately differentiated tumours were much more common in subtotal responders, indicating that poorly differentiated tumours according to the novel grading system likely do not respond well to current chemotherapeutic approaches. This finding is supported by the association of a poor differentiation according to the Cellular Dissociation Grade in pre-therapeutic biopsies with a poor response to the neoadjuvant treatment in the respective resection specimen, although this finding needs further validation in light of the rather small subset of patients where pre- and post-neoadjuvant grading data were available. Another supporting argument is the fact that high Cellular Dissociation Grade cases enrich in the population of neoadjuvantly treated when compared with primary resected tumours, which is likely due to the fact that a certain number of cases with optimal response (ypT0) are for obvious reasons “deselected” from the neoadjuvant resection cohort evaluated here.

The Cellular Dissociation Grade itself and thus the capacity of Cellular Dissociation of a given carcinoma does not seem to be strongly influenced by neoadjuvant chemoradiation, which is nicely demonstrated by the largely stable post-therapy grade in the subgroup of patients where pre-therapeutic grading data were available. This finding strongly argues against a mere selection of more poorly differentiated tumour areas of a given neoplasm by neoadjuvant therapy.

The lack of predicting factors allowing us to choose the best adjuvant therapy option not only in those patients with but also in those without neoadjuvant therapy is a continuing nuisance for us and dismal for our patients. Although it has not been prospectively tested (and thus proven) regression grade—as an indirect measure of chemotherapeutic sensitivity—might have some potential in this regard. Our data suggest that CDG might be a second factor that could add some information here, since obviously CDG high-grade cases do not respond well, thus arguing that in those cases post-surgery adjuvant treatment might also be less beneficial.

In conclusion, our study demonstrates that Cellular Dissociation Grading for oesophageal squamous cell carcinoma provides relevant independent prognostic information in the post-neoadjuvant setting. This makes Cellular Dissociation Grading the only morphology-based grading algorithm for ESCC published to date, which is able to independently contribute prognostic information in the post-neoadjuvant setting in concert with regression grade and pathological/clinical staging. In addition, evidence is mounting that Cellular Dissociation Grade holds potential as a predictor of response to conventional neoadjuvant chemotherapy.

## Supplementary information

Supplemental Material

## Data Availability

All data relevant for this study are given with the main paper including figures, tables and the supplemental files. The tissue investigated for this study is archived in the Institute of Pathology of the Technical University of Munich.
