# Supplementary material for: Post-neoadjuvant cellular dissociation grading based on tumour budding and cell nest size is associated with therapy response and survival in oesophageal squamous cell carcinoma
Source: Br J Cancer. 2019 Nov 6;121(12):1050–7. doi: 10.1038/s41416-019-0623-2 (PMC6964693; doi:10.1038/s41416-019-0623-2)
Supplement: Supplementary file 1 — Supplemental Material [file 41416_2019_623_MOESM1_ESM.docx]

**Supplementary Table 1:** Interobserver concordance between two pathologists for the Cellular Dissociation Grade.

**Supplementary Table 2:** Correlation of clinicopathological factors with cell nest size, budding activity and Cellular Dissociation Grade.

**Supplementary Table 3:** Association of Cellular Dissociation Grade with disease specific survival in multiparametric regression analysis.

**Supplementary Table 4:** Association of Cellular Dissociation Grade with overall survival in multiparametric regression analysis.

**Supplementary Figure 1:** Association of cell nest size **(A-C)** and budding activity **(D-F)** with overall, disease specific and disease-free survival.

**Supplementary Figure 2:** Association of the raw sum scores derived from tumour budding and cell nest size with overall **(A)**, disease specific **(B)** and disease free **(C)** survival.

**Supplementary Figure 3:** Association of Cellular Dissociation Grade with overall **(A)**, disease specific **(B)** and disease free **(C)** survival in the subgroup of patients with subtotal regression.


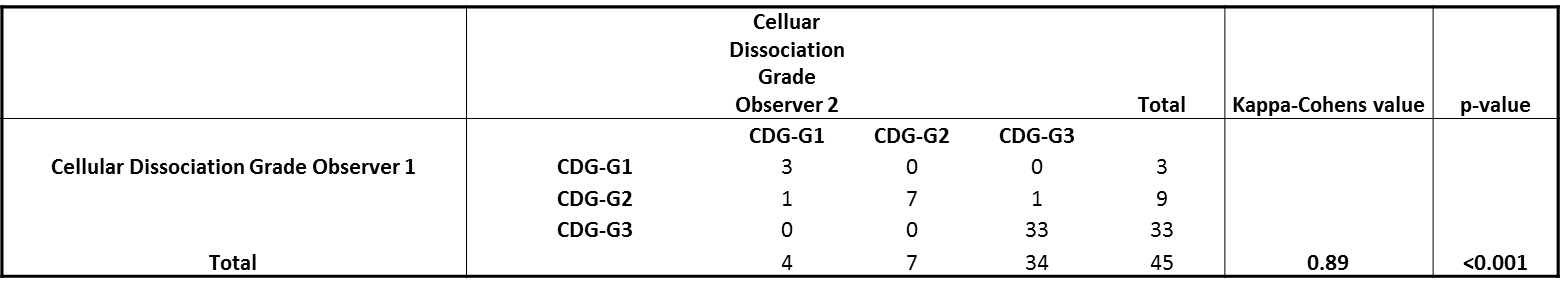


**Supplementary Table 1**

**
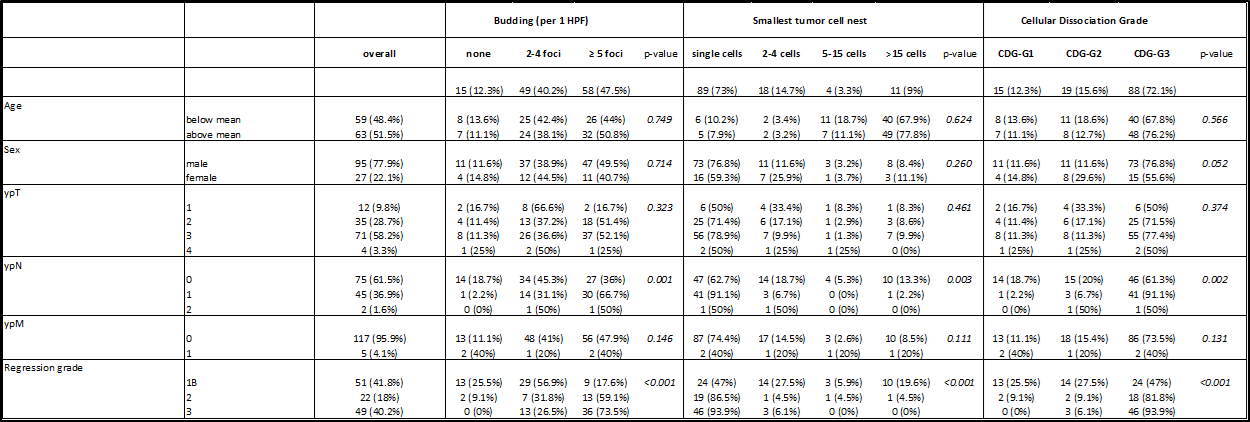
**

**Supplementary Table 2**

**
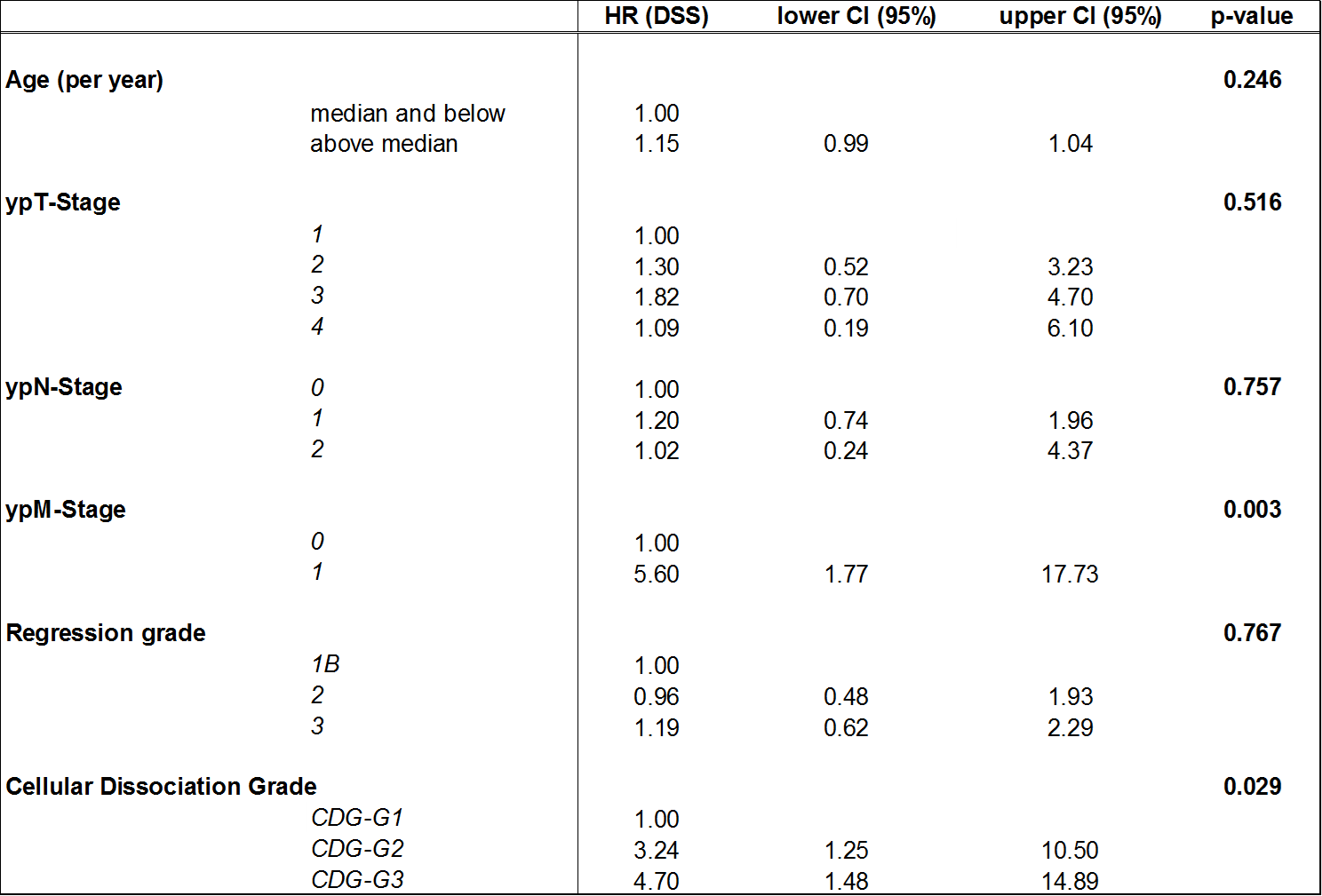
**

**Supplementary Table 3**

**
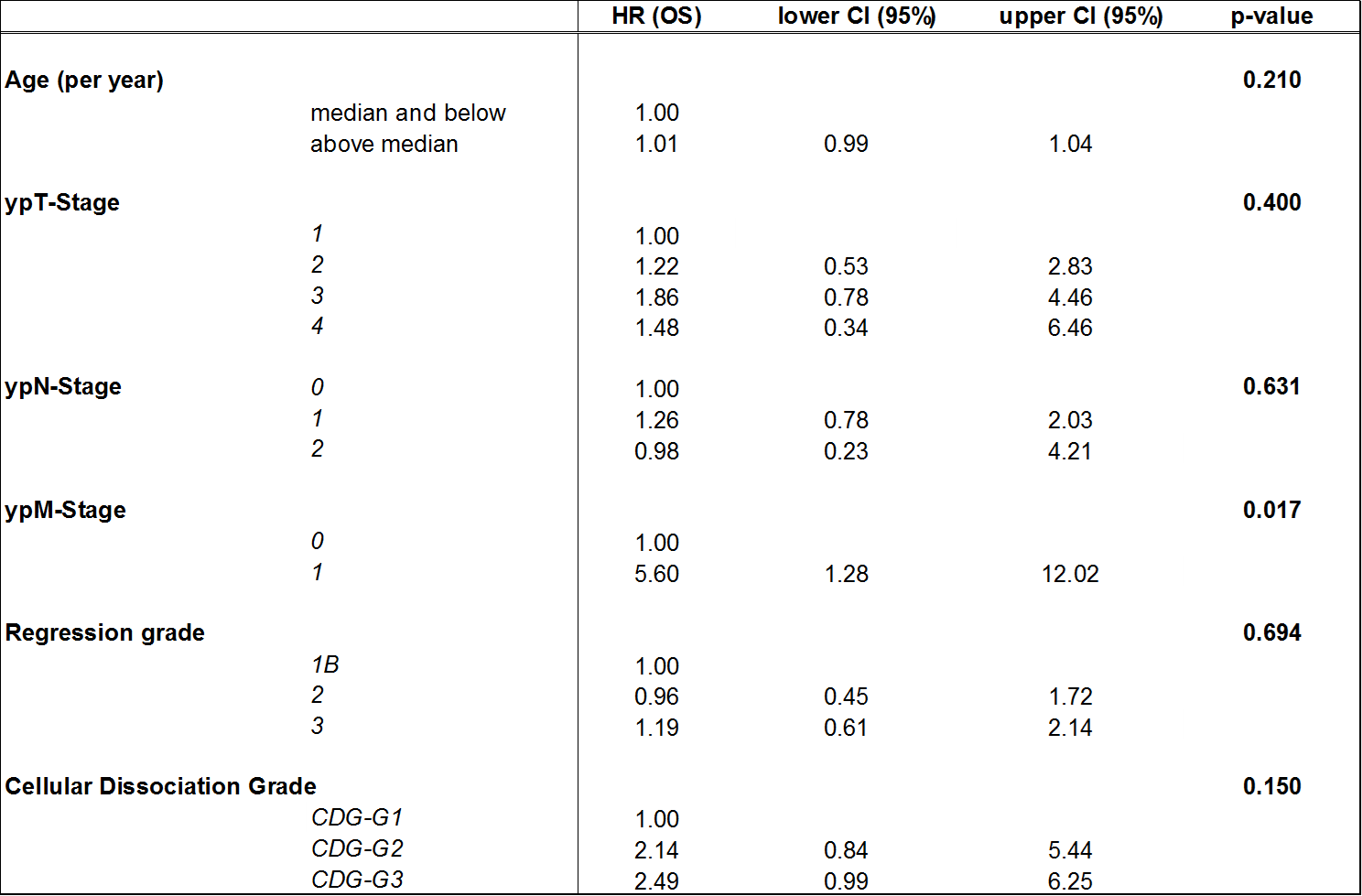
**

**Supplementary Table 4**

**

**

**Supplementary Figure 1**

**
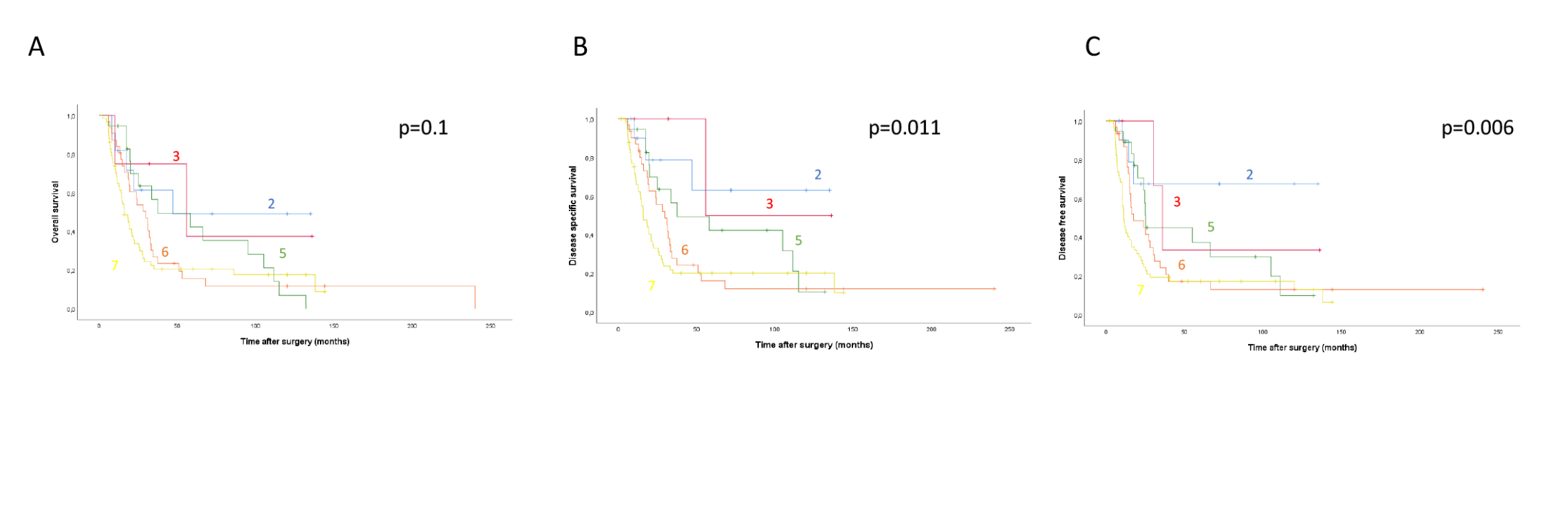
**

**Supplementary Figure 2**

**
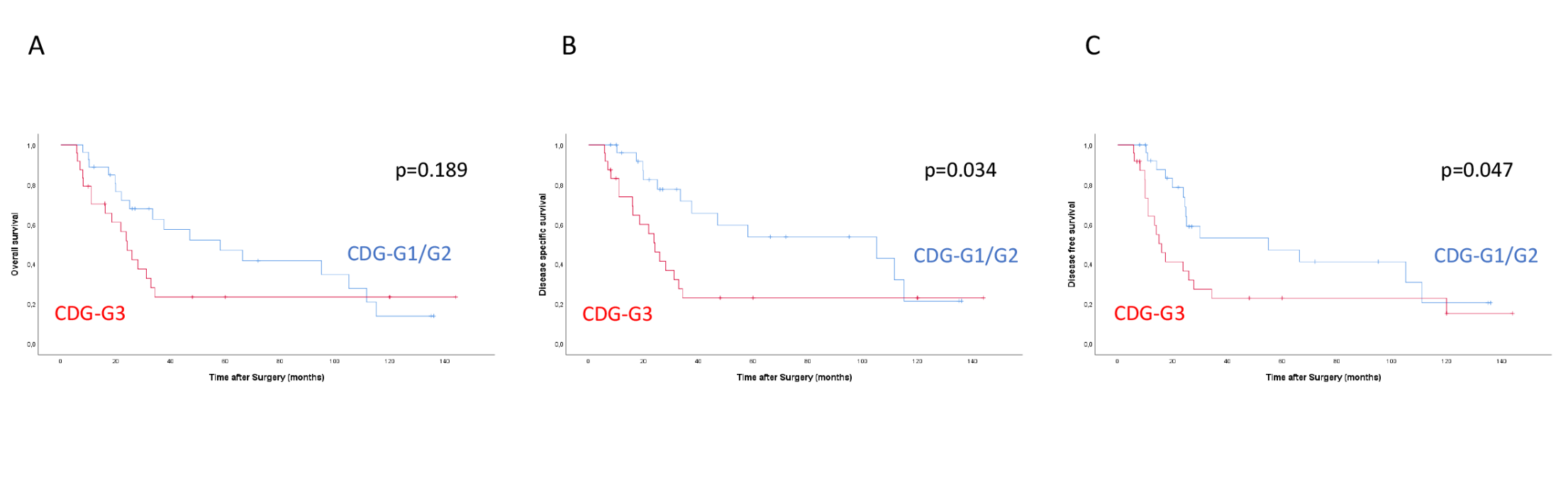
**

**Supplementary Figure 3**
